# Characterization of a novel *TFG* variant causing autosomal recessive pure hereditary spastic paraplegia

**DOI:** 10.1002/acn3.52113

**Published:** 2024-06-04

**Authors:** Cheng‐Tsung Hsiao, Tzu‐Yun Tsai, Ting‐Yi Shen, Yu‐Shuen Tsai, Yi‐Chu Liao, Yi‐Chung Lee, Pei‐Chien Tsai

**Affiliations:** ^1^ Department of Neurology Taipei Veterans General Hospital Taipei Taiwan; ^2^ Department of Neurology National Yang Ming Chiao Tung University School of Medicine Taipei Taiwan; ^3^ Department of Life Sciences National Chung Hsing University Taichung Taiwan; ^4^ Cancer and Immunology Research Center National Yang Ming Chiao Tung University Taipei Taiwan; ^5^ Brain Research Center National Yang Ming Chiao Tung University Taipei Taiwan; ^6^ Center for Intelligent Drug Systems and Smart Bio‐devices (IDS2B) National Yang Ming Chiao Tung University Hsinchu Taiwan; ^7^ The iEGG and Animal Biotechnology Research Center National Chung Hsing University Taichung Taiwan

## Abstract

**Objective:**

*TFG* mutations have previously been implicated in autosomal recessive hereditary spastic paraplegia (HSP), also known as SPG57. This study aimed to investigate the clinical and molecular features of *TFG* mutations in a Taiwanese HSP cohort.

**Methods:**

Genetic analysis of *TFG* was conducted in 242 unrelated Taiwanese HSP patients using a targeted resequencing panel covering the entire coding regions of *TFG*. Functional assays were performed using an *in vitro* cell model to assess the impact of *TFG* variants on protein function. Additionally, other representative TFG mutant proteins were examined to understand the broader implications of *TFG* mutations in HSP.

**Results:**

The study identified a novel homozygous *TFG* c.177A>C (p.(Lys59Asn)) variant in a family with adolescent‐onset, pure form HSP. Functional analysis revealed that the Lys59Asn TFG variant, similar to other HSP‐associated TFG mutants, exhibited a low affinity between TFG monomers and abnormal assembly of TFG homo‐oligomers. These structural alterations led to aberrant intracellular distribution, compromising TFG's protein secretion function and resulting in decreased cellular viability.

**Interpretation:**

These findings confirm that the homozygous *TFG* c.177A>C (p.(Lys59Asn)) variant is a novel cause of SPG57. The study expands our understanding of the clinical and mutational spectrum of *TFG*‐associated diseases, highlighting the functional defects associated with this specific *TFG* variant. Overall, this research contributes to the broader comprehension of the genetic and molecular mechanisms underlying HSP.

## Introduction

Hereditary spastic paraplegia (HSP) represents a heterogeneous group of inherited neurological disorders characterized by progressive degeneration of the motor axons in the corticospinal tracts, resulting in bilateral lower limb spasticity and weakness.^1^ HSP can be categorized into pure and complex forms, with the former caused by the isolated degeneration of the corticospinal tracts, while the complex form encompassing additional neurological system involvement. In addition to lower limb spasticity and weakness, individuals with complex HSP may present with a wide range of neurological symptoms, including ataxia, seizures, intellectual disability, muscle atrophy, extrapyramidal symptoms, visual abnormalities, and peripheral neuropathy. Up to 90 genes have been identified to be associated with HSP (https://neuromuscular.wustl.edu/spinal/fsp.html),^2^ and individual HSP‐related genes and mutations can contribute to clinical phenotypes through different mechanisms. Understanding the molecular basis underpinning these disease genes and causal mutations may facilitate the therapeutic development and provide insights into the pathophysiology of HSP.

Mutations in the *TFG* gene have been linked to various neurodegenerative diseases, including hereditary motor and sensory neuropathy (HMSN) and HSP. In 2012, a Japanese research group identified a heterozygous c.854C>T (p.(Pro285Leu)) variant in *TFG* as the cause of autosomal dominant hereditary motor and sensory neuropathy with proximal predominance (HMSN‐P).^3^ Our research team later found a heterozygous *TFG* c.806G>T (p.(Gly269Val)) variant responsible for typical Charcot–Marie–Tooth disease type 2 (CMT2) in a large Taiwanese family, characterized by autosomal dominant axonal polyneuropathy with distal predominant involvement.^4^ Furthermore, a small subset of *TFG* variants can result in autosomal recessive HSP, specifically Spastic Paraplegia type 57 (SPG57). In 2013, Beetz et al. reported that the homozygous *TFG* variant, c.316C>T (p.(Arg106Cys)), in an Indian family led to a complex form of HSP characterized by spastic paraplegia, optic nerve atrophy, and neuropathy, known as SPOAN syndrome.^5^ Intriguingly, in a consanguineous Pakistani family, another homozygous *TFG* variant, c.317G>A (p.(Arg106His)), was found to cause a childhood‐onset pure form of HSP.^6^ Based on the distribution of pathogenic variants in *TFG* discovered thus far, it can be summarized that *TFG* variants associated with neuropathy typically occur in a heterozygous pattern at the C‐terminal of the protein, while variants linked to SPG57 are the homozygous N‐terminus‐residing *TFG* variants.

In this study, we identified a novel homozygous *TFG* variant, c.177A>C, p.(Lys59Asn), in a consanguineous pedigree with autosomal recessive pure HSP. We assessed the pathogenicity of the *TFG* variant by utilizing *in vitro* cell experiments and bioinformatic analysis. Additionally, we compared the influence of TFG protein function between this variant and several previously reported pathogenic *TFG* variants.

## Patients and Methods

### Patient evaluations and ethics

A consecutive series of 242 unrelated patients with HSP were enrolled at Neurology Services of Taipei Veterans General Hospital from January 1998 to January 2022. The diagnosis of HSP is based on the manifestation of either (1) pure spastic paraplegia, (2) spastic quadriparesis with more severe and earlier involvement of lower limbs, or (3) spastic paraplegia as an early and prominent sign with involvement of multiple parts of the nervous system. Besides, other potential disease causes had been excluded through brain and spinal cord image studies and basic biochemical tests.^7^, ^8^, ^9^ This study was approved by the Institutional Review Board of Taipei Veterans General Hospital. Written informed consent was obtained from all participants prior to their involvement. A comprehensive neurological examination was conducted, including the assessment of the Spastic Paraplegia Rating Scale,^9^ to characterize the patients' clinical features. Nerve conduction studies (NCS) and electromyography (EMG) were carried out to assess the involvement of the peripheral nervous systems. Neuroimaging studies using magnetic resonance imaging (MRI) were conducted to evaluate any structural abnormalities in the brain and spinal cord.

### Genetic analyses

Genomic DNA was extracted from peripheral white blood cells. Mutational analysis of *TFG* was conducted by utilizing a targeted sequencing panel covering 76 HSP genes and the 57 other genes implicated in diseases with an HSP‐like phenotype on an Illumina HiSeq2500 (Table S1).^10^ Sequence variants within the targeted regions were called by aligning the sequenced reads to the reference Human Genome version 38 (hg38/GRCh38). The *TFG* variants were further confirmed through Sanger sequencing and named according to the reference *TFG* sequence (NM_006070.6). *In silico* prediction of the variant's pathogenicity utilized multiple bioinformatics tools, including MutationTaster,^11^ SIFT,^12^ PolyPhen‐2,^13^ PredictSNP,^14^ and CADD.^15^ Conservation scores from ConSurf^16^ and LIST‐S2^17^ were employed to assess conservation. The pathogenicity of the novel *TFG* variant was evaluated according to the guidelines of the American College of Medical Genetics and Genomics and the Association for Molecular Pathology (ACMG‐AMP).^18^

### Expression constructs

The full‐length coding region of human *TFG* was cloned into either the pcDNA 3.1/myc‐His vector (Invitrogen, Thermo Fisher Scientific, Waltham, MA, USA) or the pFLAG‐CMV‐5a vector (Sigma‐Aldrich, St. Louis, MO, USA) to create myc‐tagged or flag‐tagged wild‐type TFG expression plasmids. The *TFG* variants, c.177A>G (p.(Lys59Asn)), c.316C>T (p.(Arg106Cys)), c.317G>A (p.(Arg106His)), c.806G>T (p.(Gly269Val)), and c.854C>T (p.(Pro285Leu)), were individually introduced into the wild‐type expression plasmids using the QuikChange Site‐Directed Mutagenesis method (Stratagene, Merck, Burlington, MA, USA). The resulting constructs were verified through repeated Sanger sequencing.

### Cell culture and transfection

HEK293T or NSC‐34 cells were cultured in Dulbecco's Modified Eagle's Medium (DMEM) supplemented with 10% fetal bovine serum (FBS) at 37°C in a humidified incubator with 5% CO_2_. Neuro‐2a (N2a) neuroblastoma cells were cultured in minimal essential medium (MEM) supplemented with 10% FBS. To induce differentiation of N2a cells, they were incubated in MEM supplemented with 1 mM dibutyryl cAMP, 10 μM retinoic acid, and 1% FBS. Transient transfections were performed using Lipofectamine 2000 (Invitrogen).

### CRISPR/Cas9 knockout of *TFG* in HEK293T, neuro‐2a, and NSC‐34 cells

To deplete endogenous *TFG* in cells using CRISPR/Cas9 technology, *TFG* gRNA (target sequence in Exon 2: ATCCTGGAGTCCACCATGAA for human *TFG*; ATCCTGGACTCCACAATGAA for mouse *Tfg*) in pCas‐Guide‐EF1a‐GFP vector (OriGene, Rockville, MD, USA) was transfected into cells (Fig. S1A). After 3 days, cells were seeded as single colonies in 96‐well plates. Once individual colonies had formed, they were isolated and cultured in 6‐well plates until cell numbers were sufficient for western blot analyses (Fig. S1B). Genomic DNA was isolated from cells using QuickExtract solution (Epicentre, Madison, WI, USA), following the manufacturer's instructions. The modified region was amplified using designed validation primers and sequenced to determine the clonal genotype (Fig. S1A).

### Cross‐linking of oligomeric TFG proteins

To examine the oligomeric state of TFG, lysates from TFG‐overexpressing cells were cross‐linked by incubating them with various concentrations of Bis (sulfosuccinimidyl) suberate sodium salt (BS3) in conjugation buffer (100 mM sodium phosphate, 0.15 M NaCl) for 30 min. The cross‐linking reactions were then quenched by incubating with 50 mM Tris HCl, pH 7.5. Subsequently, the reaction mixtures were analyzed using western blotting.

### Co‐immunoprecipitation (co‐IP) analysis

To investigate the self‐assembly ability of TFG proteins, co‐IP analysis was performed to assess the binding affinity between TFG molecules. In brief, HEK293T cells were co‐transfected with flag‐ and myc‐tagged TFG constructs. Cell extracts were prepared using Pierce IP Lysis Buffer (Thermo Fisher Scientific). With the exception of 10% of the sample volume used for input, the lysates and anti‐flag antibody‐conjugated Dynabeads (Invitrogen) were incubated overnight at 4°C. After washing, the target proteins were eluted by boiling in 1X SDS loading buffer at 95°C for 5 min, and these eluates were subsequently analyzed by western blotting.

### Immunofluorescence studies

Cells were fixed in 4% paraformaldehyde, followed by permeabilization with 0.2% Tween‐20 and blocking with 1% BSA. The cells were then incubated overnight at 4°C with primary antibodies. The bound primary antibodies were detected using Alexa‐conjugated secondary antibodies. Imaging was conducted using an Olympus FluoView FV10i confocal laser scanning fluorescence microscopy system with a 60X oil immersion objective (Olympus, Tokyo, Japan).

### Investigating protein secretory efficiency through the *Gaussia* luciferase reporter assay

*Gaussia* luciferase (Gluc) is commonly used to monitor the protein secretory pathway.^19^, ^20^ Secreted Gluc activity in the culture medium from co‐transfected cells expressing both Gluc and *Firefly* luciferase (Fluc) serves as an indicator of protein secretory efficiency. To evaluate this, cells were co‐transfected with plasmids expressing Gluc (pCMV‐Gaussia Luc; Thermo Fisher Scientific), Fluc (pCMV‐Red Firefly Luc; Thermo Fisher Scientific), and either TFG wild‐type, variants, or an empty vector. After 48 h of transfection, conditioned medium was collected to measure Gluc activity, which was then normalized to the corresponding Fluc activity in the cell lysate for each sample set.

### Cell viability assay

Cells were seeded onto 96‐well culture plates and transfected with the specified *TFG* constructs. After 48 h, cell viability was assessed using the Cell Counting Kit‐8 (CCK‐8) assay (Sigma‐Aldrich) following the manufacturer's instructions.

### Neurite outgrowth assays

Neurite outgrowth assays were performed using the Neurite Outgrowth Staining Kit (Thermo Fisher Scientific). N2a cells in a 96‐well plate were transfected with the specified *TFG* constructs. After 16 h of transfection, cells were induced to differentiate for 48 h and fixed with 4% paraformaldehyde. Neurite outgrowth and cell viability were assessed by staining with fluorescent dyes for the cell membrane and viability. The samples were then analyzed using a fluorescence microplate reader.

## Results

### Identification of the *TFG* variant

**Figure 1:**
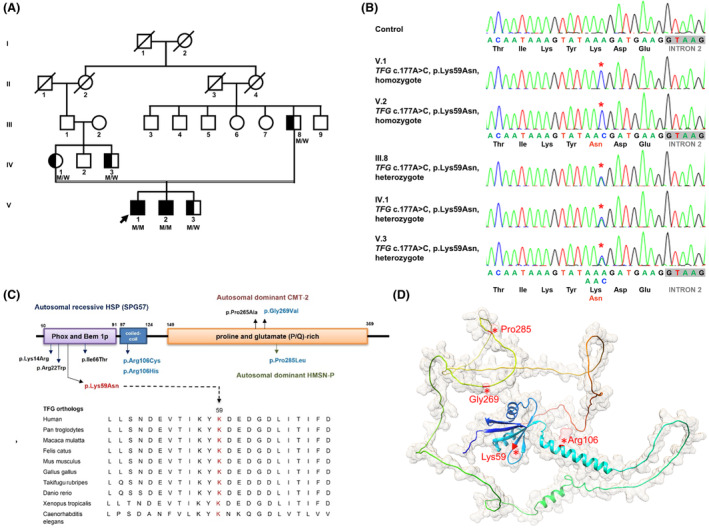
Identification of the homozygous *TFG* missense variant in the pedigree with pure hereditary spastic paraplegia. (A) Pedigree of the HSP patient harboring the *TFG* p.(Lys59Asn) variant. The proband is indicated by an arrow. “M” denotes the mutant TFG allele, while “W” represents the wild‐type allele. Squares and circles correspond to males and females, respectively. Filled and open symbols indicate affected and unaffected individuals, respectively. Half‐filled symbols represent carriers of the heterozygous mutation without displaying the disease phenotype. A slash indicates deceased individuals. (B) Electropherograms of *TFG* Exon 2 in a wild‐type control, the HSP‐manifested siblingship, and their unaffected carrier family members. The altered nucleotides are highlighted with red star symbols. (C) Schematic representation of the functional domains of the TFG protein and the comparison of the amino acid sequences of TFG orthologous proteins from various species. Several known pathogenic *TFG* variants are labeled, with the *TFG* p.(Lys59Asn) variant identified in this study highlighted in red. Additional variants examined in this study are marked in blue. Alignment of multiple TFG orthologs demonstrates conservation of the K59 residue from human to *Caenorhabditis elegans*. (D) Visualization of the AlphaFold‐predicted TFG protein (PDB: AF‐Q92734‐F1) structure on the UCSF Chimera software (V1.14). The loci of *TFG* variants characterized in this study are labeled in red.

Among the 242 unrelated HSP patients, only one patient was identified to carry a *TFG* variant, which was the homozygous *TFG* c.177A>C (p. (Lys59Asn)) (Fig. 1A,B). The patient manifested pure HSP and originated from a consanguineous family. Further analysis revealed the same homozygous variant in one of his brothers, who also presented with pure HSP. Four other individuals in the family carrying a heterozygous *TFG* c.177A>C (p.(Lys59Asn)) variant did not exhibit any neurological deficits. This *TFG* variant had not been previously reported and was absent in the Genome Aggregation Database (gnomAD v4.0.0; http://gnomad.broadinstitute.org/) and the Taiwan Biobank database (http://www.twbiobank.org.tw/), which contains 1517 genomes from healthy Taiwanese individuals. The pathogenicity of *TFG* p.(Lys59Asn) was also supported by multiple bioinformatics prediction tools (Table 1). According to the ACMG‐AMP guidelines,^18^ *TFG* c.177A>C (p.(Lys59Asn)) meets several criteria, including cosegregation with HSP in a recessively inherited pattern within the family (PP1 criterion), absence in the population databases (PM2), and its deleterious effect supported by multiple predicting programs (PP3) and functional studies (PS3). Collectively, *TFG* c.177A>C (p.(Lys59Asn)) is classified as a likely pathogenic variant.

**Table 1:** The *TFG* coding variant identified in the study.

			Conservation		Predicted pathogenicity				
Gene	cDNA change	Protein change	ConSurf^a^	LIST‐S2^b^	Mutation Tester	SIFT^c^	PolyPhen‐2	PredictSNP	CADD^d^
*TFG*	c.177A>C	p.(Lys59Asn)	8	0.92	Deleterious	Deleterious (0.2)	Probably damaging	Deleterious	24.7

^a^ ConSurf scores range from 1 to 9, indicating the conservation level of amino acid residues.

^b^ The LIST‐S2 score, the closer it is to 1, indicates a higher level of conservation.

^c^ The SIFT score ranges from 0.0 (deleterious) to 1.0 (tolerated).

^d^ CADD, Combined Annotation‐Dependent Depletion. A variant with a CADD score >20 indicates that it belongs to the top 1% most deleterious variants in the genome, and >30 places it among the top 0.1%.

### Case presentation

The proband (V‐1) harboring the homozygous c.177A>C (p.(Lys59Asn)) *TFG* variant was born to the consanguineous parents who were neurologically healthy and were genotyped for the heterozygous *TFG* c.177A>C (p.(Lys59Asn)) variant (Fig. 1A,B). Patient V‐1 had an unremarkable birth history and achieved normal developmental milestones during childhood. He began to experience gradual worsening of gait characterized by intermittent stiffness of the legs and frequent falls since age 15 years. These symptoms persisted and worsened after age 31 years when he suffered from fatigue after walking long distances and had difficulty walking downstairs. Despite these symptoms, he did not require walking aids and retained normal function of daily activities. Neurological examinations at age 36 revealed normal muscle strength but lower limbs spasticity, resulting in limited dorsi‐ and plantar‐flexion of the ankles and mild spasticity in the hips and knees. The deep tendon reflex (DTR) examination revealed bilateral brisk knee jerks and ankle clonus, with normal tendon reflex at the upper limbs. Although he was able to walk without assistance, a scissor gait was evident during the evaluation (Video S1). No sensory abnormalities or sphincter dysfunction were reported. The spastic paraplegia rating scale (SPRS) yielded a score of 13/60. The MRI studies excluded structural abnormalities at the cervical and thoracic levels. Electrophysiological evaluations, including NCS and EMG, revealed normal sensory and motor conduction, as well as normal electromyographic activities.

The proband's younger brother (V‐2), who harbored homozygous *TFG* c.177A>C (p.(Lys59Asn)) variant, had suffered from progressive gait abnormalities and frequent falls since age 15 years. He reported that the leg stiffness became more prominent in cold weather, leading to difficulty going downstairs and mild fatigue while walking upstairs. Neurological examinations at age 34 years revealed lower limbs spasticity, bilateral brisk knee and ankle jerks, and a spastic gait (Video S2). The SPRS score was 9/60 at age 34 years. His spine MRI demonstrated unremarkable findings. Electrophysiological examinations further revealed normal results. In contrast, neurological examinations of the family members carrying the heterozygous *TFG* c.177A>C (p.(Lys59Asn)) variant were all normal, including the proband's parents (III‐8 and IV‐1) at age 60 and 55 and his youngest brother (V‐3) at age 25 (Fig. 1A).

### Impact of *TFG* variants on protein oligomerization

To assess the functional alterations induced by the *TFG* p.(Lys59Asn) variant, our investigation began by examining the effects associated with Lys59Asn TFG in the absence of wild‐type TFG. CRISPR‐Cas9 was initially employed to knock out TFG expression in HEK293T, N2a, and NSC‐34 cells for two primary reasons: First, *TFG* c.177A>C (p.(Lys59Asn)) causes autosomal recessive HSP in its homozygous status, and second, TFG protein is ubiquitously expressed in neuronal and non‐neuronal cells. This approach aimed to establish a cellular model for analyzing homozygous variants linked to recessive disorders. To comprehensively understand the effects of mutant proteins and elucidate potential pathological mechanisms of *TFG* p.(Lys59Asn), our functional studies included other representative pathogenic *TFG* variants: p.(Arg106Cys) and p.(Arg106His) linked to SPG57, and p.(Gly269Val) and p.(Pro285Leu) associated with CMT2 and HSMN‐P (Fig. 1C,D).

**Figure 2:**
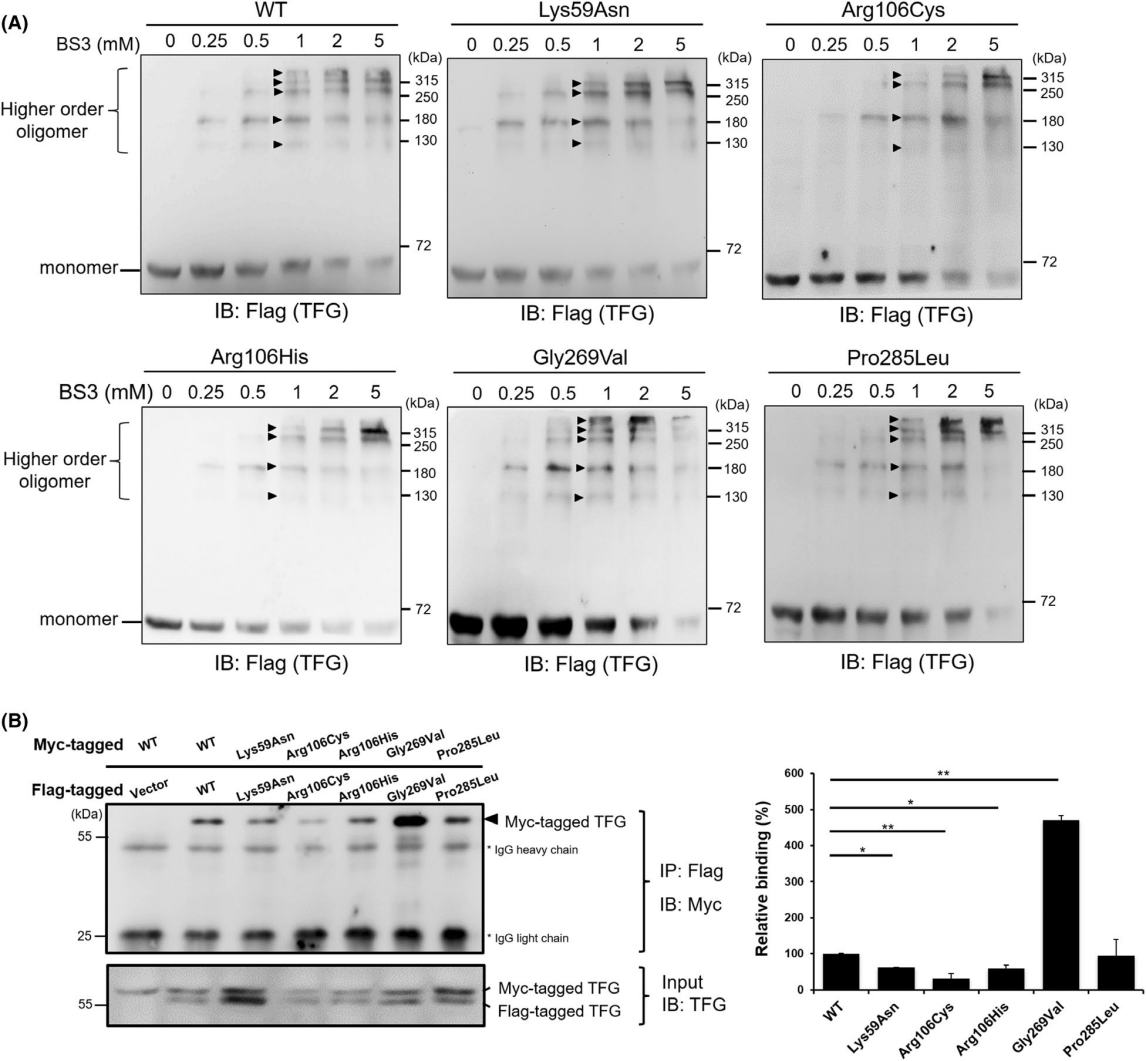
Defective oligomerization and self‐assembly of HSP‐associated TFG mutants. (A) Oligomeric structure of TFG proteins *in vitro*. TFG variant‐expressing cells underwent cross‐linking reactions with varying concentrations of BS3. The resulting products were analyzed through western blotting. The HSP‐related TFG Lys59Asn, Arg106Cys, and Arg106His mutants exhibited the absence of the highest‐tier oligomeric products. Arrowheads indicate the positions of bands where higher‐order oligomers appear. (B) Evaluation of the self‐assembly capacity of TFG proteins using co‐immunoprecipitation assays. Cells were co‐transfected with myc‐tagged and flag‐tagged TFG expression plasmids, followed by lysis 48 h posttransfection. The interaction among TFG protein molecules was demonstrated by co‐immunoprecipitation pull‐downs of myc‐tagged TFG using an anti‐flag antibody against flag‐tagged TFG. The degree of binding between TFG protein molecules is graphically presented in a quantitative statistical chart on the right‐hand panel. Values are shown as means ± SEM of three independent experiments (*p < 0.05; **p < 0.01). “WT” denotes wild‐type.

Previous study has shown that TFG forms an octameric structure capable of self‐association to generate larger polymers.^21^ TFG p.(Lys59Asn) induces alterations in the Phox and Bem1p (PB1) domain of TFG, known to govern self‐association and homo‐oligomer formation, as evidenced by yeast two‐hybrid experiments.^22^ To investigate whether p.(Lys59Asn) affects TFG protein homo‐oligomerization, we conducted an *in vitro* cross‐linking assay with the BS3 chemical. In Fig. 2A, untreated wild‐type TFG proteins showed a single band at approximately 57 kDa, representing the monomeric form. In the presence of BS3, the intensity of this band decreased, and at 1 mM BS3, five new bands with molecular masses exceeding 100 kDa emerged. With a gradual increase in BS3 concentration (1–5 mM), the intensity of these new bands increased, indicating the formation of homo‐oligomers of TFG proteins *in vitro*. However, the assembly states of Lys59Asn, Arg106Cys, and Arg106His TFG mutant proteins lacked the highest band (>315 kDa), whereas the wild‐type TFG protein and the two neuropathy‐associated Gly269Val and Pro285Leu TFG variants displayed five distinct bands representing the complete oligomeric form of TFG proteins.

To further validate the impact of the SPG57‐associated *TFG* variants on protein assembly, we conducted co‐immunoprecipitation experiments to examine the self‐interaction abilities of these disease‐related TFG proteins within the *TFG*‐knockout HEK293T cells. We co‐transfected cells with myc‐tagged and flag‐tagged TFG proteins and found that the three TFG mutants associated with SPG57, including Lys59Asn, Arg106Cys, and Arg106His TFG, all exhibited weaker self‐interaction abilities than the self‐interaction abilities between wild‐type TFG molecules (Fig. 2B). Notably, among these TFG mutants, Arg106Cys TFG displayed the weakest self‐interaction ability. In contrast, the Gly269Val TFG, which is associated with CMT2, exhibited an enhanced protein self‐interaction ability. The protein self‐interaction ability of the Pro285Leu TFG, which is associated with HMSN‐P, was similar to that of wild‐type TFG.

**Figure 3:**
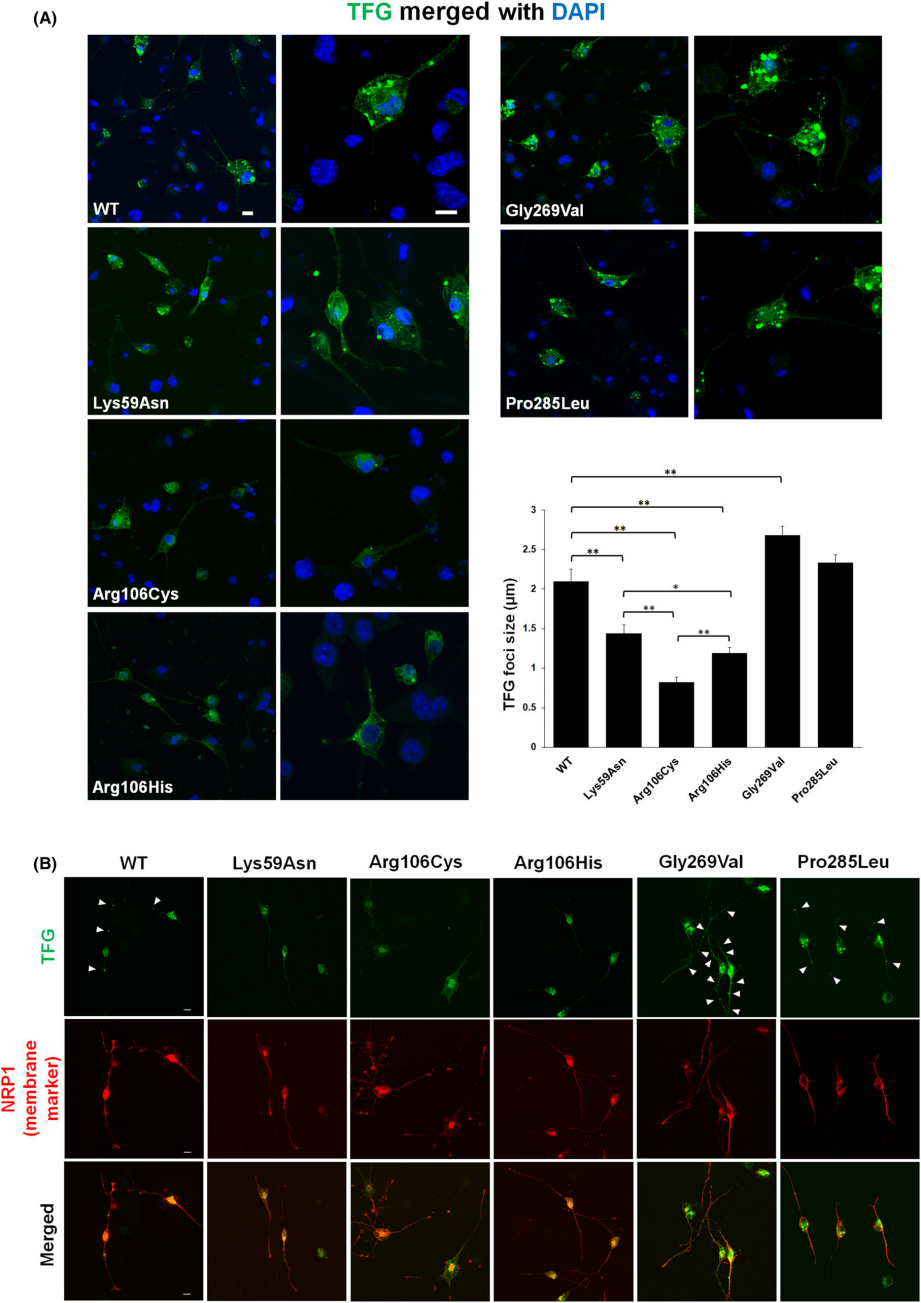
Expression and subcellular localization of the TFG variants. (A) Confocal fluorescence images of transfected N2a cells (*Tfg* knockout) labeled with an Alexa 488‐conjugated anti‐flag antibody to visualize TFG (shown in green). Cell nuclei were counterstained with DAPI (blue). The quantitative graph in the bottom right panel shows the analysis of the size of TFG foci formed by various TFG mutants in N2a cells (*Tfg* knockout). Error bars represent mean ± SEM. *p < 0.05, **p < 0.01. (B) Confocal fluorescence images of differentiated N2a cells (*Tfg* knockout) transfected with individual *TFG* constructs (labeled with a flag antibody, green) and a neurite membrane marker (highlighted by the NRP1 antibody, red). Cell nuclei were stained with DAPI (blue). Arrowheads indicate the positions of distinct high‐intensity TFG oligomer structures in the neurite. Scale bar: 10 μm.

Next, we utilized immunofluorescence techniques to observe the intracellular distribution of wild‐type or mutant TFG proteins overexpressed in the *Tfg*‐knockout N2a cells. As illustrated in Fig. 3A, the wild‐type TFG protein exhibited a granular aggregation pattern within the cytoplasm, highlighting its oligomeric nature. In contrast, the protein expression of Lys59Asn, Arg106Cys, and Arg106His TFG appeared to be less concentrated and distributed extensively throughout the cytoplasm with significantly reduced granular aggregation. Among them, Arg106Cys TFG exhibited the most dispersed pattern with the smallest protein foci, followed by Arg106His and Lys59Asn TFG. Moreover, the Gly269Val TFG protein shows larger protein foci accumulations, consistent with our prior observations.^4^ On the other hand, the Pro285Leu TFG displayed a protein appearance similar to the wild‐type TFG.

### Expression and localization of TFG variants in neuronal cells

We evaluated the expression of *TFG* variants in differentiated neuronal cell lines by transfecting various *TFG* variants into *Tfg*‐knockout N2a cells. Following differentiation, immunofluorescence staining was utilized to visualize TFG protein expression and localization in differentiated neurons. As shown in Fig. 3B, the wild‐type TFG exhibited a granular aggregation pattern within the cytoplasm and extended into the neurites. However, the protein expression of p.(Lys59Asn), p.(Arg106Cys), and p.(Arg106His) variants predominantly distributed within the cytoplasm of neurons, with minimal observation in the neurites. Variants associated with neuropathy, including p.(Gly269Val) and p.(Pro285Leu), exhibit a distribution pattern similar to that of the wild type. Overall, these results indicate that the three SPG57‐associated variants disrupt TFG protein assembly, leading to impaired oligomerization and altered distribution within both the cytoplasm and neurites.

### Effects of *TFG* variants on protein transport and cell viability

**Figure 4:**
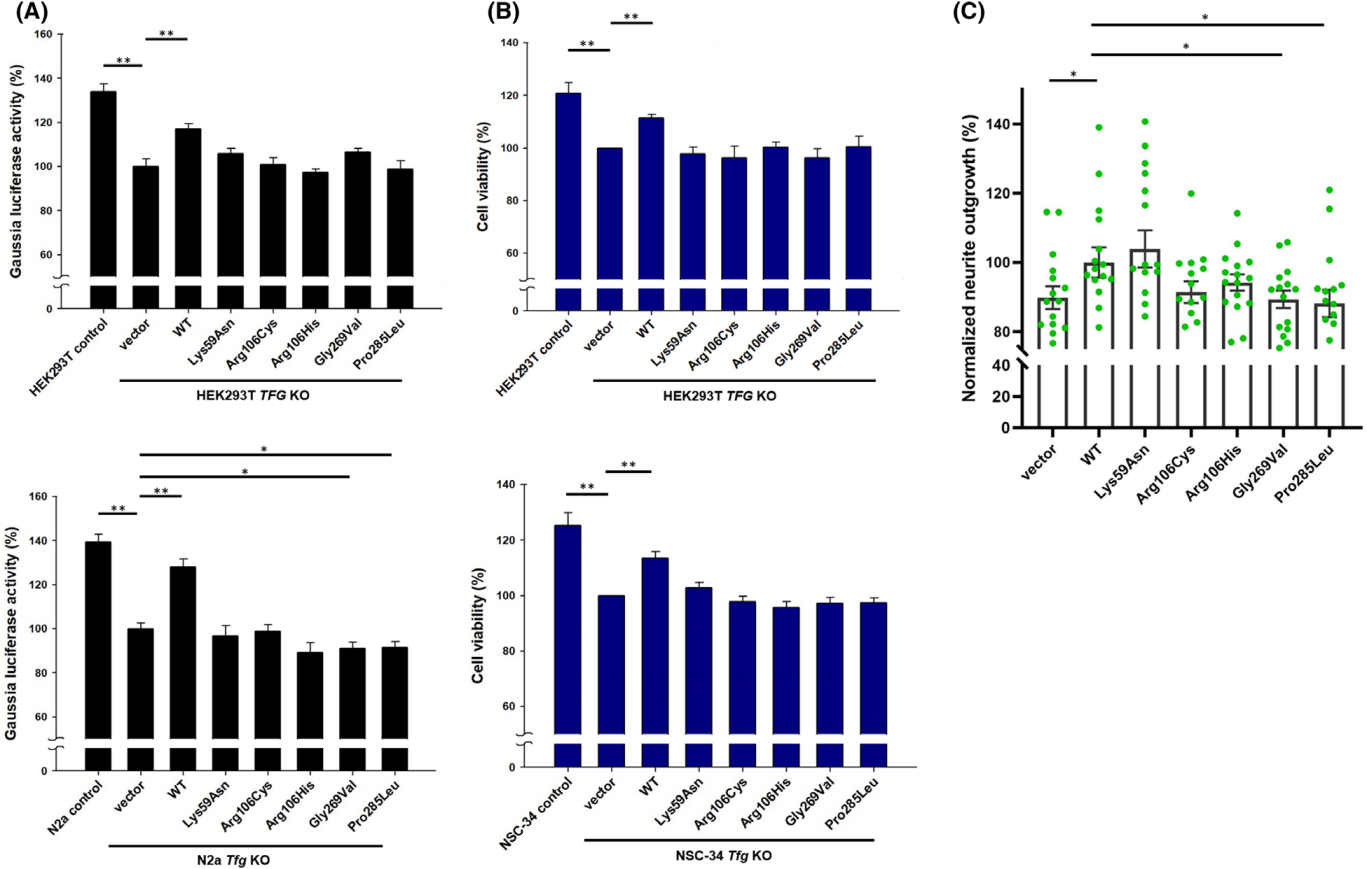
*In vitro* functional analysis of the TFG variants (A) Quantitative analysis of secreted *Gaussia* Luciferase (Gluc) activities in the culture medium of co‐transfected HEK293T or N2a cells expressing Gluc, *Firefly* Luciferase (Fluc), and TFG plasmids. The measured Gluc activities were normalized to the corresponding Fluc activities in cell lysates to evaluate protein secretory efficiency. The Gluc activity percentage of *TFG* or *Tfg* knockout cells transfected with an empty vector was set as 100% for comparison. Results are presented as means ± SEM from 9 (HEK293T) or 12 (N2a) independent transfections (*p < 0.05; **p < 0.01). “KO” indicates knockout. (B) Assessment of cell viability in HEK293T or NSC‐34 cells expressing TFG using the Cell Counting Kit‐8. The viability percentage of *TFG* or *Tfg* knockout cells transfected with an empty vector was set as 100% for comparison. The presented values represent means ± SEM from 6 (HEK293T) or 18 (NSC‐34) independent transfections. (C) Neurite outgrowth analysis in N2a cells expressing TFG mutants. N2a cells (*Tfg* knockout) were transfected with the *TFG* construct or an empty vector, cultured under differentiation conditions, and stained with a cell membrane fluorescent dye and a cell viability indicator fluorescent dye. Normalized neurite outgrowth indicates the relative fluorescence units of the cell membrane dye normalized to the corresponding cell viability indicator dye. The presented values represent means ± SEM from 16 independent transfections.

Previous studies have emphasized TFG's role in the COPII‐mediated vesicle transport pathway.^21^, ^23^, ^24^ In this study, we evaluated the impact of these *TFG* variants on protein transport by co‐transfecting *TFG* variants with two reporter plasmids in *TFG*‐knockout HEK293T or N2a cells. In *TFG*‐knockout cells transfected with an empty vector, we observed significantly reduced protein secretion efficiency compared to the original cell control without *TFG*‐knockout (Fig. 4A). Introducing the wild‐type TFG into *TFG*‐knockout cells significantly improved protein secretion efficiency. However, cells expressing other external TFG variants were unable to rescue protein secretion efficiency, indicating that all five *TFG* variants disrupt cellular protein transport. We also performed a CCK‐8 cell viability assay to assess the effect of *TFG* variants on cell viability. This revealed significantly reduced cell viability in *TFG*‐knockout vector control cells compared to the original cells without *TFG*‐knockout (Fig. 4B). The introduction of external wild‐type TFG into *TFG*‐knockout cells notably increased cell viability, but the expression of other external TFG mutant variants failed to rescue cell viability, demonstrating that all five disease‐associated *TFG* variants can influence cell viability.

### Influence of TFG variant expression on neurite outgrowth

We assessed the influence of TFG variant expression on neurite outgrowth in *Tfg*‐ knockout N2a cells using the Neurite Outgrowth Staining Kit. As illustrated in Fig. 4C, when compared to wild‐type TFG expression, the introduction of neuropathy‐associated TFG variants (i.e., Gly269Val and Pro285Leu) led to a substantial reduction in neurite outgrowth. In the contrast, the three variants linked to SPG57 showed no significant difference from the wild‐type.

## Discussion

The significance of *TFG*‐associated HSP has been increasingly recognized due to the growing number of *TFG* variants identified in HSP patients. These patients predominantly exhibit an autosomal recessive inheritance pattern, denoted as SPG57,^5^, ^6^, ^25^, ^26^, ^27^ or less frequently as an autosomal dominant subtype.^28^ In this study, we identified a novel homozygous *TFG* c.177A>C (p.(Lys59Asn)) variant in a pair of siblings presenting with adolescent‐onset pure spastic paraplegia within a consanguineous family in Taiwan. This finding introduces *TFG* p.(Lys59Asn) as the seventh variant associated with HSP.

Our study revealed that the p.(Lys59Asn) variant in the PB1 domain of the TFG protein, as well as the p.(Arg106Cys) and p.(Arg106His) variants in the coiled‐coil domain, all resulted in reduced intermolecular affinity, leading to abnormal protein assembly and disruption of TFG's high‐ordered oligomeric structure. Previous studies have also noted the impact of the p.(Arg106Cys) variant on TFG intermolecular assembly,^5^, ^29^, ^30^ and another SPG57‐associated variant, *TFG* p.(Arg22Trp), in the PB1 domain, compromised TFG oligomerization.^25^ The PB1 and coiled‐coil domains are known to play crucial roles in TFG's oligomerization and interactions with other proteins.^22^, ^31^, ^32^, ^33^, ^34^ Therefore, the proper folding and homo‐oligomerization of these two N‐terminal domains are essential for maintaining TFG's normal structure. Altering certain conserved amino acids within the PB1 or coiled‐coil domain may hinder the self‐assembly function of TFG through different mechanisms, thereby affecting TFG's structure and function.

Furthermore, in this study, we observed that the p.(Lys59Asn) variant, along with two other SPG57‐associated variants, exhibited altered cellular localization and distribution. Wild‐type TFG typically accumulates at the ER exit site (ERES) within the cytoplasm, where its oligomeric structure acts as a scaffold protein facilitating the transport of COPII‐coated vesicles.^21^, ^23^, ^24^ Additionally, TFG is known to aggregate and distribute in the axons and dendrites of nerve cells, supporting the cycling transport of endosome cargoes.^35^ However, our study revealed that SPG57‐associated TFG mutants exhibited reduced aggregation at the ERES, ultimately affecting the intracellular protein transport efficiency. In differentiated neuronal cells, while SPG57‐associated TFG mutant proteins did not significantly affect differentiation and neurite growth, the granule structure of TFG was visibly smaller, and these proteins were sparsely aggregated in neurites. A previous study has also noted that the p.(Arg106Cys) variant resulted in fewer TFG foci in neurites of primary rat cortical neurons and disrupted the normal endosomal trafficking of gephyrin to synapses.^35^ Therefore, it is suggested that TFG p.(Lys59Asn) may also influence TFG's role in endosome cargo transport.

Moreover, remarkably, there is a positive correlation between the severity of alterations in molecular affinity, structural size, and intracellular distribution among TFG molecules and the clinical severity of these variants. The Lys59Asn TFG protein showed the mildest impact on molecular affinity, oligomeric structure size, and intracellular distribution, corresponding to the mildest clinical symptoms. In contrast, the Arg106Cys TFG showed the smallest oligomeric structure and the most diffuse distribution, and was nearly absent in neurites, aligning with the most severe clinical symptoms. We employed the machine learning‐based software MutPred2^36^ to predict the effects of these three SPG57‐associated variants. The results indicated that the p.(Arg106Cys) variation, located in the coiled‐coil domain, may potentially induce an “altered coiled coil” effect (*p* value = 0.002), while p.(Arg106His) did not exhibit this effect, suggesting that p.(Arg106Cys) has a more significant impact on the protein structure. On the other hand, the p.(Lys59Asn) variant might lead to a “loss of ubiquitylation at K59” (*p* value = 0.01). In the results presented in Fig. S2, we also observed that the protein steady‐state expression level of Lys59Asn TFG is indeed higher than that of the wild type, possibly due to the variant affecting its protein degradation regulation. Furthermore, the steady‐state expression levels of Arg106Cys TFG are the lowest, followed by Arg106His. The TFG levels inside the cells may also play a role in determining the clinical phenotype's severity. However, these speculations require further experimental validation.

Additionally, SPG57 mutations at different sites yield distinct clinical manifestations. The *TFG* p.(Lys59Asn) variant identified in this study is the sole PB1 domain variant known to cause pure HSP. Patients with this variant typically experience symptom onset in their late adolescence and exhibit milder clinical symptoms than other SPG57‐associated variants. Conversely, three other PB1 domain variants, p.(Lys14Arg), p.(Arg22Trp), and p.(Ile66Thr), result in early onset complex HSP.^25^, ^26^, ^27^ Notably, p.(Ile66Thr) shares similarities with *TFG* p.(Arg106Cys) and is associated with optic nerve atrophy. Therefore, mutations at different sites may induce varying degrees of structural and functional impairments, potentially affecting interactions with distinct proteins, disrupting diverse downstream signaling pathways, and leading to a wide array of clinical symptoms.

In addition to SPG57‐associated *TFG* variants, we also examined two variants, p.(Gly269Val) and p.(Pro285Leu), linked to CMT2 and HMSN‐P, for functional comparisons. Interestingly, these two variants, responsible for peripheral neuropathy, do not disrupt TFG molecular assembly and oligomerization. Instead, the *TFG* p.(Gly269Val) variant significantly increases the molecular affinity among TFG molecules, leading to larger TFG protein aggregates within cells. Our previous research indicated that the *TFG* p.(Gly269Val) variant increases the tendency for protein deposition, exhibiting a dominant negative effect, resulting in the accumulation of insoluble protein aggregates and a reduction in functional TFG levels within cells.^4^ Furthermore, the *TFG* p.(Gly269Val) and p.(Pro285Leu) variants do not alter the distribution of TFG within the cytoplasm or neurites; however, they have a more pronounced impact on neurite outgrowth. These findings highlight significant differences in the mechanisms responsible for structural or functional abnormalities induced by *TFG* variants associated with peripheral neuropathies compared to those related to SPG57.

Combining the functional results of this study, the pathogenic mechanisms underlying the three SPG57‐associated variants, TFG Lys59Asn, Arg106Cys, and Arg106His proteins, appear to lean toward a loss of function. TFG plays a critical role in various membrane system‐mediated protein transport processes,^21^, ^23^, ^24^, ^35^, ^37^ which are particularly crucial for maintaining the normal physiology of neurons compared to other cell types. Many pathogenic genes in HSP are involved in membrane modeling, including proteins such as Spastin M1, REEP1, Atlastin, Spastizin, Spatacsin, and Strumpellin, which are associated with the ER, endosomes, autophagosomes, and lysosomes. Any functional defects in these proteins can easily lead to neuronal and axonal pathological conditions, subsequently causing the disease.

In conclusion, this study identifies a novel SPG57‐associated *TFG* variant, c.177A>C (p.(Lys59Asn)) and delineates the relevant functional disturbance of TFG proteins. These findings expand the clinical knowledge and mutational spectrum of SPG57 and provide insights into the molecular pathogenic mechanisms of HSP.

## Author Contributions

Cheng‐Tsung Hsiao and Pei‐Chien Tsai contributed to the conception and design of the study. Tzu‐Yun Tsai, Ting‐Yi Shen, and Yu‐Shuen Tsai conducted the experiments and acquired the data. Cheng‐Tsung Hsiao, Yi‐Chung Lee and Yi‐Chu Liao recruited patients and conducted clinical assessments. Cheng‐Tsung Hsiao, Yi‐Chung Lee, Yi‐Chu Liao, and Pei‐Chien Tsai analyzed and interpreted the data, and drafted the manuscript. Pei‐Chien Tsai supervised the entire research process. All authors read and approved the final version of the manuscript.

## Conflict of Interest

The authors disclose no conflicts of interest.

## Supporting information


Data S1.

Video S1.

Video S2.
