# Supplementary material for: Characterization of a novel TFG variant causing autosomal recessive pure hereditary spastic paraplegia
Source: Ann Clin Transl Neurol. 2024 Jun 4;11(7):1909–20. doi: 10.1002/acn3.52113 (PMC11251477; doi:10.1002/acn3.52113)
Supplement: Supplementary file 1 — Data S1. [file ACN3-11-1909-s002.docx]

**The supplementary information file contains supplementary Table S1, Figure S1, Figure S2 and legends for Video S1 and Video S2.**

| **Table S1.** The list of HSP-associated genes targeted in the sequencing panel | | | | | | |
| --- | --- | --- | --- | --- | --- | --- |
| *AAAS* | *ARSI* | *DARS* | *GJC2* | *MFN2* | *RETREG1* | *TDP2* |
| *ABCD1* | *ARX* | *DARS2* | *HSPD1* | *MLC1* | *RNF170* | *TECPR2* |
| *ABHD12* | *ATL1* | *DDHD1* | *IBA57* | *MTHFR* | *RPIA* | *TFG* |
| *ACD* | *ATP13A2* | *DDHD2* | *IDUA* | *MTPAP* | *RTN2* | *TINAGL1* |
| *ACO2* | *ATP2B4* | *DSTYK* | *IFIH1* | *NEFL* | *RUBCN* | *TPP1* |
| *AFG3L2* | *ATP6AP2* | *ELOVL4* | *KANK1* | *NIPA1* | *SACS* | *TTC19* |
| *AIMP1* | *ATRX* | *ENTPD1* | *KCNA2* | *NKX6-2* | *SELENOI* | *UBA2* |
| *ALDH18A1* | *B4GALNT1* | *EPRS* | *KIDINS220* | *NT5C2* | *SETX* | *UCHL1* |
| *ALDH3A2* | *BICD2* | *ERCC2* | *KIF1A* | *OPA3* | *SIL1* | *USP8* |
| *ALS2* | *BSCL2* | *ERLIN1* | *KIF1C* | *PAH* | *SLC16A2* | *VAMP1* |
| *AMPD2* | *C19orf12* | *ERLIN2* | *KIF5A* | *PDYN* | *SLC25A15* | *VCP* |
| *AP4B1* | *CACNA1G* | *EXOSC3* | *KLC2* | *PGAP1* | *SLC2A1* | *VPS37A* |
| *AP4E1* | *CAPN1* | *FA2H* | *L1CAM* | *PLP1* | *SLC30A10* | *WASHC5* |
| *AP4M1* | *CCT5* | *FARS2* | *LMNB1* | *PNPLA6* | *SLC33A1* | *WDR48* |
| *AP4S1* | *CLCN2* | *FOLR1* | *LYST* | *POLR3A* | *SPART* | *WWOX* |
| *AP5Z1* | *CPT1C* | *GAD1* | *MAG* | *PSEN1* | *SPAST* | *ZFR* |
| *ARG1* | *CYP27A1* | *GALC* | *MARS* | *RAB3GAP2* | *SPG11* | *ZFYVE26* |
| *ARL6IP1* | *CYP2U1* | *GBA2* | *MARS2* | *REEP1* | *SPG21* | *ZFYVE27* |
| *ARSA* | *CYP7B1* | *GFAP* | *MECP2* | *REEP2* | *SPG7* | *ZNF592* |


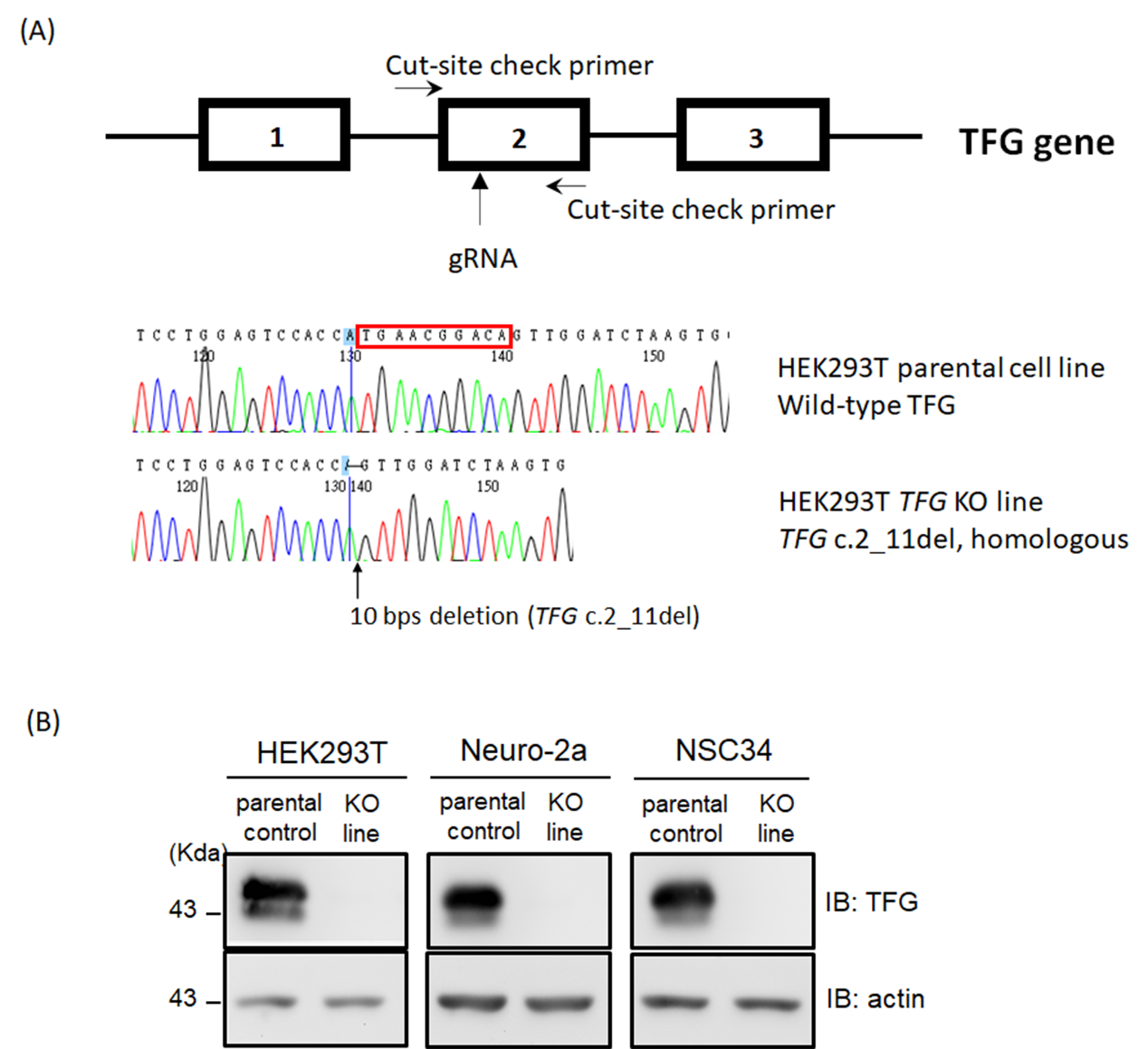


**Figure S1.** Validation of *TFG* knockout (KO) cell lines. (A) The upper panel illustrates the location of the guide RNA (gRNA) target designed in this study, targeting exon 2 of the human or mouse *TFG* gene, along with the primer positions used for Sanger sequencing validation. The lower panel shows the electropherograms of sequencing validation for HEK293T *TFG* knockout clone, demonstrating a 10-base pair deletion (enclosed by the red square) generated by CRISPR-Cas9 technology near the start codon ATG of exon 2 of the *TFG* gene, resulting in the homozygous *TFG* c.2_11del mutation. (B) Western blotting validation results for HEK293T, Neuro-2a, and NSC34 *TFG* knockout clones showing the absence of endogenous TFG protein expression in all *TFG* knockout clones.


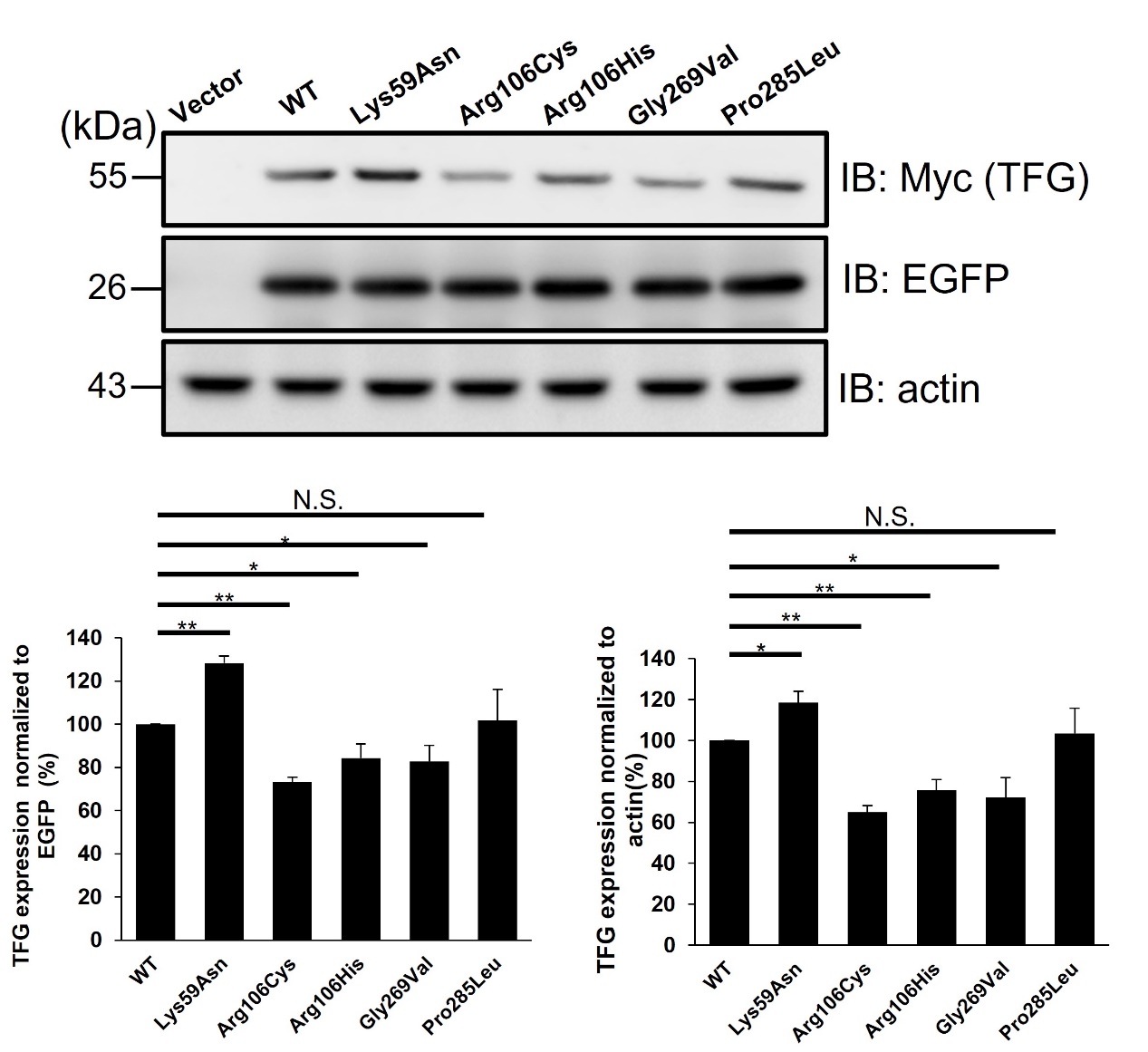


**Figure S2.** Representative Western blot analysis of TFG steady-state expression in the HEK293T *TFG* knockout cells co-transfected with plasmids expressing either wild-type (WT) TFG, one of the TFG variants, or an empty vector, along with a transfection efficiency control, enhanced green fluorescent protein (EGFP). Densitometric quantification is presented below the blots, with error bars representing the standard error of the mean (SEM) from three independent experiments (*, *p* < 0.05; **, *p* < 0.01; N.S., not significant). EGFP or actin densitometry was used as the transfection or loading control to normalize the densitometry of TFG.

**Supplementary Video Legends**

**Video S1:** The neurological examination conducted for the proband (V-1) at his age of 36 years revealed the following: (1) hyper-reflexia of bilateral patellar reflexes and bilateral ankle clonus; (2) increased muscle tone in the bilateral knee joints; and (3) a spastic gait characterized by difficulty in knee extension and slight ankle adduction with weight-bearing on the toes while walking.

**Video S2:** The neurological evaluation performed for the proband's younger sibling (V-2) when he aged 34 years demonstrating: (1) bilateral brisk knee jerks and ankle clonus; and (2) spasticity in the lower extremities accompanied by a spastic gait.
